# Temperature-Dependence of Rubber Hyperelasticity Based on the Eight-Chain Model

**DOI:** 10.3390/polym12040932

**Published:** 2020-04-17

**Authors:** Xintao Fu, Zepeng Wang, Lianxiang Ma, Zhaoxuan Zou, Qingling Zhang, Xinxin Guan

**Affiliations:** College of Electromechanical Engineering, Qingdao University of Science and Technology, Qingdao 266061, China; fuxintao_qust@163.com (X.F.);

**Keywords:** filled rubber, temperature-dependent, eight-chain model, hyperelasticity, finite element analysis (FEA)

## Abstract

Rubber-based materials are widely used in a variety of industrial applications. In these applications, rubber components withstand various loading conditions over a range of temperatures. It is of great significance to study the mechanical behavior of vulcanized rubber at different temperatures, especially in a range of high temperatures. The temperature dependence of the constitutive behavior of filled rubber is important for the performance of the rubber. However, only a few constitutive models have been reported that investigate the stress–temperature relationship. In this paper, based on an analysis of experimental data, the effects of temperature on the hyperelastic behaviors of both natural rubber and filled rubber, with different mass fractions of carbon black, were studied. The regulation of stress and strain of natural rubber and filled rubber with temperature was revealed. In addition, an eight-chain model that can reasonably characterize the experimental data at different temperatures was proved. An explicit temperature-dependent constitutive model was developed based on the Arruda-Boyce model to describe the stress–strain response of filled rubber in a relatively large temperature range. Meanwhile, it was proved that the model can predict the effect of temperature on the hyperelastic behavior of filled rubber. Finally, the improved Arruda-Boyce model was used to obtain the material parameters and was then successfully applied to finite element analysis (FEA), which showed that the model has high application value. In addition, the model had a simple form and could be conveniently applied in related performance test of actual production or finite element analysis.

## 1. Introduction

Due to its superior comprehensive properties, rubber has been widely used, such as for tires, motor bases, footwear, pipes, transmission belts, etc. Rubber products are often used in harsh working conditions, and in a wide range of temperatures [1,2]. Seasonal temperature changes can cause significant variations in the mechanical properties of rubber. Therefore, the impact of temperature on the mechanical properties of rubber must be considered.

Excessive temperature of elastomeric components may be caused by either the surrounding environment or inherent heating due to internal dissipation during cyclic loading. In addition, the low thermal conductivity of rubber exacerbates this situation [3]. The degradation and dynamic instability of the rubber elastomer due to cyclic loading overheating have been investigated in the literature [4].

Although the mechanical responses of filled and unfilled rubber have been characterized at room temperature [5,6,7,8,9], the effects of temperature on the mechanical response of rubber materials in a certain deformation range, such as 150% strain, have rarely been studied. Especially, the operation temperatures of rubber are usually in a wide range depending on the application [10]. Most tensile tests on rubber-like materials have only been performed at room temperature [11,12]; therefore, it is necessary to investigate the properties of these materials at various temperatures other than room temperature. In addition, a change in temperature affects the interaction between the filler and rubber matrix [13]. From previous studies, filled rubber exhibited more complex temperature-dependent characteristics, and the performance of rubber material significantly depended on temperature [14,15,16,17,18]. It is important to develop a constitutive model which can clearly reveal and describe the temperature characteristics of elastomeric components [19].

The basic characteristics of the stress–strain behavior of rubber are usually described by the large strain elastic model, which is based on two methods [20,21]. In the first method, the change of configurational entropy of randomly oriented long molecular chains is considered based on the framework of statistical mechanics [22,23]. These models provide a good tool for predicting the elastic behavior of large strains and have a minimum number of physically related material parameters. The second method is phenomenological, which is based on the invariance of stretches and the framework of continuum mechanics. This phenomenological method has been successfully used to simulate large strain elastic responses of unfilled and filled rubber [24,25].

Some thermomechanical models have been proposed, including a model to evaluate the influence of temperature on the mechanical properties of filled rubber [3,15,18]. However, these models have various defects when it comes to characterizing the mechanical behavior of filled rubber. In summary, based on the Arruda-Boyce model [26], which is also known as the eight-chain model, a hyperelastic mechanical model with explicit temperature parameters was proposed. The model can accurately describe the hyperelastic behavior of tire rubber at different temperatures.

In this work, a series of uniaxial tensile tests of natural rubber and four different types of filled tire rubber were performed at different temperatures. Based on the accurate test data, the effects of temperature on the mechanical properties of tire rubber were discussed, and the ability of the eight-chain model to describe the experimental data of filled rubber at different temperatures was confirmed. The Arruda-Boyce model was extended to an explicit temperature-dependent form by considering the relationship between Arruda-Boyce model parameters and ambient temperature. The evaluation results showed that the model can accurately reveal the influence of temperature on the hyperelastic behavior of tire rubber.

The tensile process of rubber specimen was successfully reproduced by using the improved eight-chain model in the finite element software ABAQUS, and the simulation data were compared with the experimental data. The results showed that the improved eight-chain model can ideally represent the experimental data and can easily be applied in engineering.

## 2. Experimental Section

### 2.1. Experimental Materials

Four types of rubber materials filled with different contents of carbon black were used. The rubber matrix was natural rubber and the filled carbon black was N234. Among the four rubber formulations, only the amount of filled carbon black was different. The filling mass fractions of carbon black in the four types of rubber, i.e., C00, C20, C40, and C60, were 0 phr, 20 phr, 40 phr, and 60 phr, respectively. The codes and formulas of the four types of rubber used in the tests are shown in Table 1. Natural rubber was obtained from Shandong Haoshun Chemical Co., Ltd., Jinan, Shandong, China. CB N234 was obtained from Shanghai Auman Chemical Co., Ltd., Shanghai, China. The other agents were used as purchased. Stearic acid, zinc oxide, sulfur, accelerator NS, and antioxidant 4020 were industrial grade products.

### 2.2. Sample Preparation

The mixing was carried out with an open roll mixing mill (Type: S(X)-160A, Shanghai No. 1 rubber machinery, Shanghai, China). Firstly, NR was masticated on a two roll mill at for 5 min. Then, sulfur, accelerator NS, antioxidant, stearic acid, and zinc oxide were added in order over the following 15 min. Finally, carbon black was added to mix the natural rubber composite well, which took about 10 min. The vulcanizations were carried out at 150 °C for 20 min under 10 MPa.

The prepared samples had a dumbbell Type 2 shape according to ISO 37-2017. The thickness of the rubber specimen was 2 mm. A uniaxial tensile test was performed using an MTS CMT4104 microcomputer-controlled electronic universal testing machine with a temperature box. The force value and displacement accuracy were both 0.5, the temperature control accuracy was ±1 K, and the gauge length of the displacement sensor was 20 mm. The fixture used a double eccentric wheel clamp, RA-4-1, which is a special tensile clamp for rubber. Because of the low thermal conductivity of rubber, the temperature must be accurately controlled. Specifically, in order to ensure the specimen reached the required test temperature, the temperature control box was stabilized for 10 min before the tensile test, after reaching the test temperature. In order to eliminate the Mullins effect (stress softening effect of the rubber material) [27,28,29], before the formal test, each sample was modulated by repeating the loading–unloading cycle in a certain deformation range 10 times. The purpose of this step was to reproduce the stress state of the tire rubber more accurately. During the modulation process, 150% strain was selected as the modulation strain, and the modulation temperature was set to 281 K. After the modulation, the rubber specimens were allowed to stand for more than 24 h so that the elastic deformation was completely restored and the performance was stable. The experiment under each condition was repeated at least 4 times, and the average obtained served as the final experimental result.

## 3. Uniaxial Tensile Test Results of Filled Rubber at Different Temperatures

The deformation of rubber is generally less than 100% in engineering strain, and 150% strain can be applied to most working conditions. Thus, in the uniaxial tensile test, 150% strain was used to characterize the mechanical properties of rubber materials.

**Figure 1:**
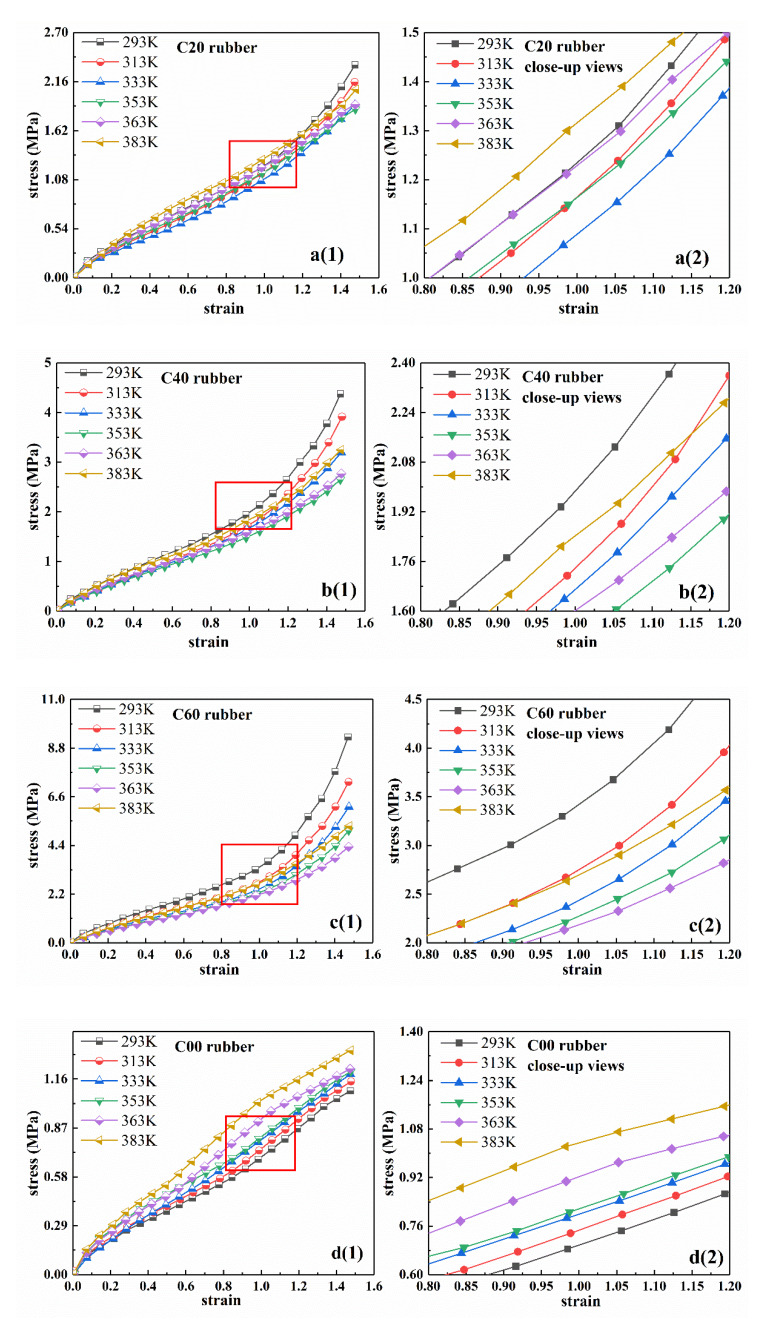
Uniaxial tensile stress–strain curves of four types of rubber compounds at different temperatures, (**a1**–**d1**) are the uniaxial tensile experimental data of four types of rubber. (**a2**–**d2**) are the corresponding local closed-up views.

Figure 1 shows the relationship between nominal stress and nominal strain of four types of rubber in Table 1: C20, C40, C60, and C00. The temperatures were 293 K, 313 K, 333 K, 353 K, 363 K, and 383 K, respectively. In order to show the difference in stress-strain curves among the four rubber samples at different temperatures more clearly, the corresponding close-up views of these curves are shown in Figure 1a2–d2. From the figures, the hyperelastic behavior of rubber material had obvious temperature dependence in the deformation range of 150%. For CB-filled rubber C20, C40, and C60, as the temperature increased, the stress–strain curve first dropped and then increased after the temperature exceeded a certain critical value. In other words, with the increase of temperature, the rubber specimen first softened and then hardened after the temperature exceeded a certain critical value. For unfilled natural rubber, C00, the stress–strain curve always increased with the increase of temperature and there was no transition point in the curve.

**Figure 2:**
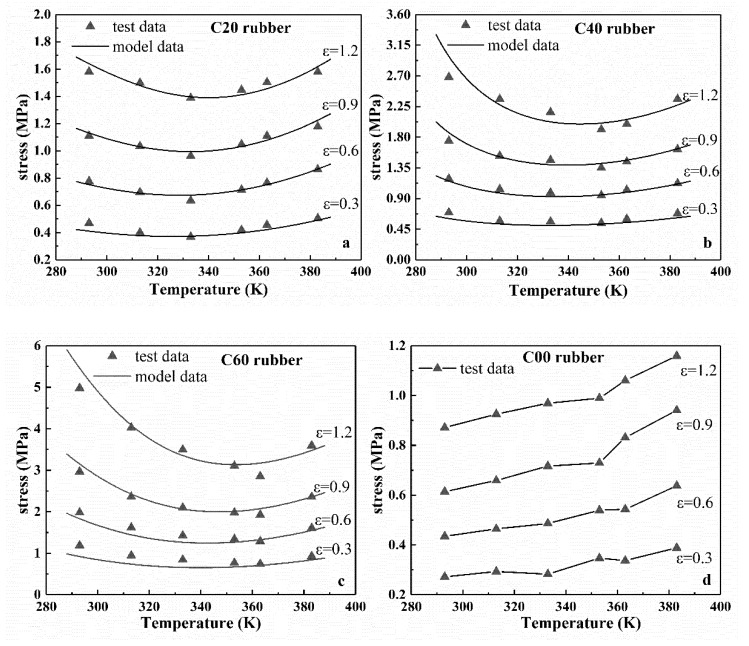
Stress–temperature curves at different constant strains for four types of rubber specimens. (**a**–**c**) are the Equations (8)’s model curves and experimental data of CB-filled rubber C20, C40, and C60. (**d**) is the experimental data of unfilled natural rubber C00.

Figure 2a–c shows the stress-temperature relationship of rubber specimens at certain strains of 0.3, 0.6, 0.9, and 1.2. From the results, as the temperature increased, the stress under constant strain for the three different types of filled rubber (i.e., C20, C40, and C60) first decreased and then increased. Therefore, with the increase of temperature, the filled rubber first softened and then gradually hardened when the temperature reached a certain value. In addition, the temperature at the transition point was different for the rubber with different carbon black filling mass fractions. From the results, the filling amount of carbon black had great influence on the mechanical properties of rubber. For the unfilled rubber C00, at constant strain, the stress increased nearly linearly with the increase of temperature, as shown in Figure 2d. These results are consistent with the conclusions of the corresponding stress-strain curves described above.

## 4. The Capability of Eight-Chain Model in Characterizing Temperature-Dependence in a Certain Deformation Range

Based on the uniaxial tensile data of filled rubber at different temperatures, the capability of the eight-chain model in characterizing the temperature-dependence was investigated. According to the Langevin statistic of Kuhn and Grün [30], the entropy of a single molecular chain ($s_{chain})$ can be obtained as follows:

(1) $$s_{chain}=k\left[c-N\left(\frac{r_{chain}}{Nl}\beta +\ln\frac{\beta }{\sinh\beta }\right)\right]$$

where c is a constant, k is the Boltzmann constant, β is the inverse of the Langevin, *N* is the number of the segments in a single chain, l is the length of each segment of chain, and $r_{chain}$ is the length of the current chain during the stretching process. $\beta ={ℒ}^{-1}\left[r_{chain}/Nl\right]$, where the Langevin function is defined as ℒ[β]=cothβ−(1/β).

The deformation work is proportional to the change of entropy in the stretching process of molecular chains, which can be expressed by the length of chains. Then, the expression of the strain energy density function of the single chain can be obtained.

(2) $$W=nk\theta N\left(\frac{r_{chain}}{Nl}\beta +\ln\frac{\beta }{\sinh\beta }\right)-\theta c^{’}$$

where *n* is the number of chains per unit volume, θ is the absolute temperature, *c’* is a combination of constants, and the other terms are the same as previously defined.

**Figure 3:**
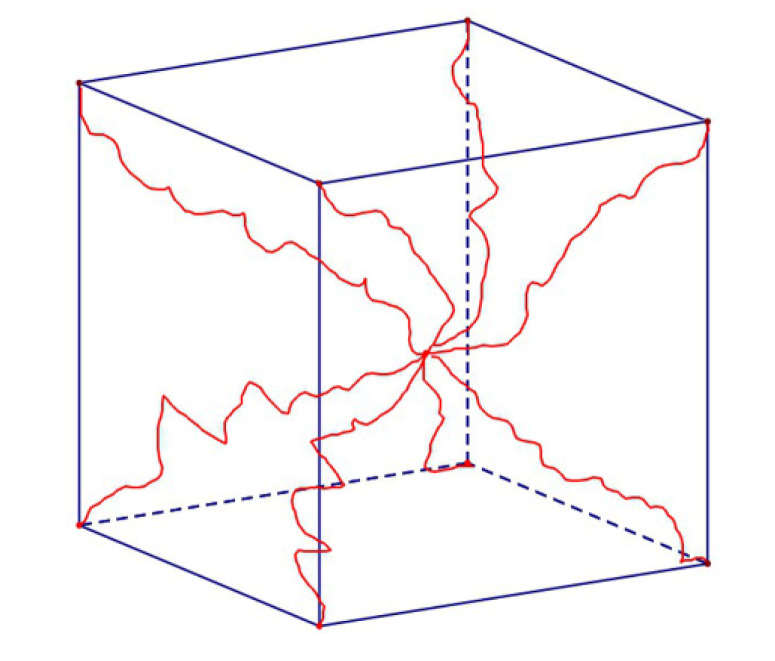
Eight-chain rubber elasticity model.

As shown in Figure 3, the eight-chain model consists of eight molecular chains extending from the center of a cube to the eight vertices of the cube [26,31]. The expression of the strain energy density function of the eight-chain model can be inferred:

(3) $$W_{8}=nk\theta \sqrt{N}\left[\beta \lambda_{chain}+\sqrt{N}\ln\left(\frac{\beta }{\sinh\beta }\right)\right]$$

where $\lambda_{chain}=r_{chain}/r_{0}\;\;$, which is defined as the drawing ratio of the chain in the stretching process of rubber. $r_{0}$ is the initial chain length.

The eight-chain model can be expanded to the form of the Taylor series (only the first five terms are retained):

(4) $$W_{8}=nk\theta \left[\frac{1}{2}\left(I_{1}-3\right)+\frac{1}{20N}\left(I_{1}^{2}-9\right)+\frac{11}{1050N^{2}}\left(I_{1}^{3}-27\right)+\frac{19}{7000N^{3}}\left(I_{1}^{4}-81\right)+\frac{519}{673750N^{4}}\left(I_{1}^{5}-243\right)\right]$$

where $I_{1}$ is the first invariant of stretches.

According to the formula given by Rivlin [32,33], the relationship between nominal stress f and stretch λ under uniaxial tension is:

(5) $$f=2\left(\lambda -\frac{1}{\lambda^{2}}\right)\left[\frac{\partial W}{\partial I_{1}}+\frac{1}{\lambda }\frac{\partial W}{\partial I_{2}}\right]$$

From Equations (4) and (5), the nominal stress expression of the eight-chain model under uniaxial tension can be obtained as follows [34]:

(6) $$f=C_{R}\left(\lambda -\frac{1}{\lambda^{2}}\right)\left[3+\frac{3}{5N}\left(\lambda^{2}+\frac{2}{\lambda }\right)+\frac{33}{175N^{2}}{\left(\lambda^{2}+\frac{2}{\lambda }\right)}^{2}+\frac{57}{875N^{3}}{\left(\lambda^{2}+\frac{2}{\lambda }\right)}^{3}+\frac{1557}{67375N^{4}}{\left(\lambda^{2}+\frac{2}{\lambda }\right)}^{4}\right]$$

$\;\;C_{R}$ can be defined as $C_{R}\;$= nkθ. Two parameters, $C_{R}$ and N, need to be determined from experimental data.

**Figure 4:**
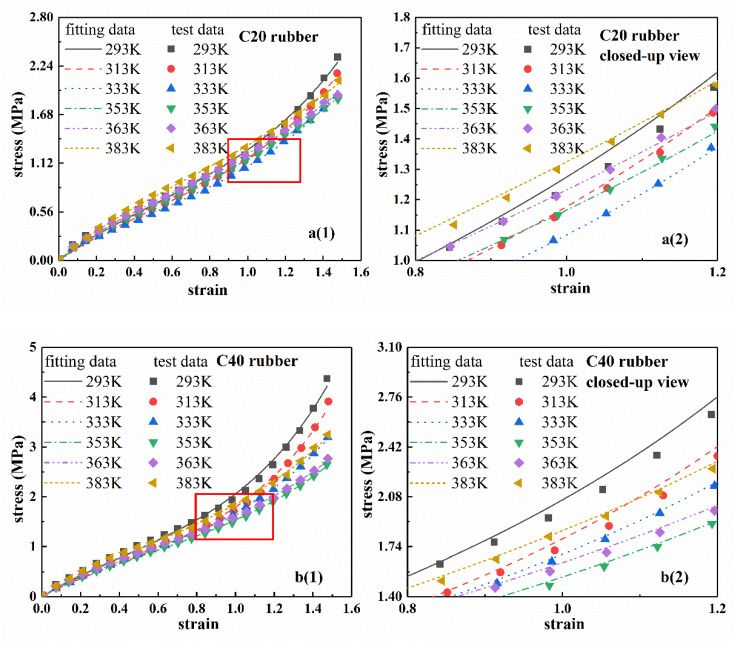

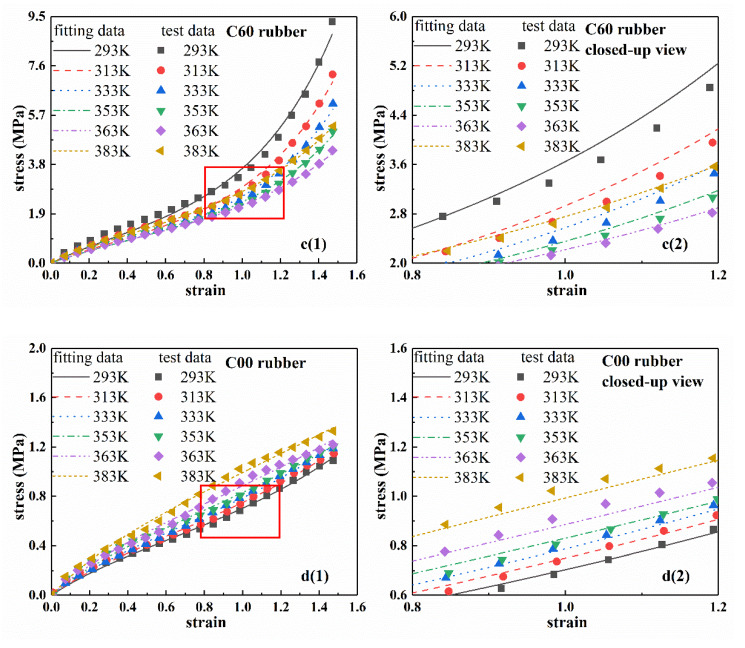
Stress–strain fitting curves of four types of rubber compounds at different temperatures based on eight-chain model. (**a1**–**d1**) are the fitting curves and experimental data of four types of rubber. (**a2**–**d2**) are the corresponding local closed-up views.

According to Equation (6) and test results, the stress–strain curves of rubber materials at different temperatures were fitted by Levenburg-Marquardt non-linear least square method [35]. The results are shown in Figure 4, where the fitting results and test data of C20, C40, C60, and C00 are successively provided. In addition, in order to further observe the fitting results, the local enlarged views are shown in Figure 4a2–d2. Table 2 shows the fitting parameters of the eight-chain model for each rubber at different temperatures. From Figure 4, the fitting curve agreed well with the experimental curve, which indicated that the eight-chain model can accurately characterize the stress–strain process of the rubber at different temperatures.

## 5. Explicit Expression of Temperature-Dependence in Eight-Chain Model

At present, in the eight-chain model, the effects of temperature on the characterization results of rubber at different temperatures is not explicitly analyzed. Thus, the eight-chain model cannot directly reflect the dependence of rubber elasticity on temperature. The fitting curves obtained by the eight-chain model at different temperatures were in good agreement with the experimental results. In addition, for different tested rubber, the parameters *Cr* and *N* at different ambient temperatures were obtained. Therefore, temperature was implicitly considered in the stress-strain relationship in Arruda-Boyce model. To facilitate the application of the model, it is necessary to extend Arruda-Boyce model to a form with explicit temperature dependence. Therefore, the relationship between material parameters and temperature in the eight-chain model was explored using the macroscopic phenomenological theory.

**Figure 5:**
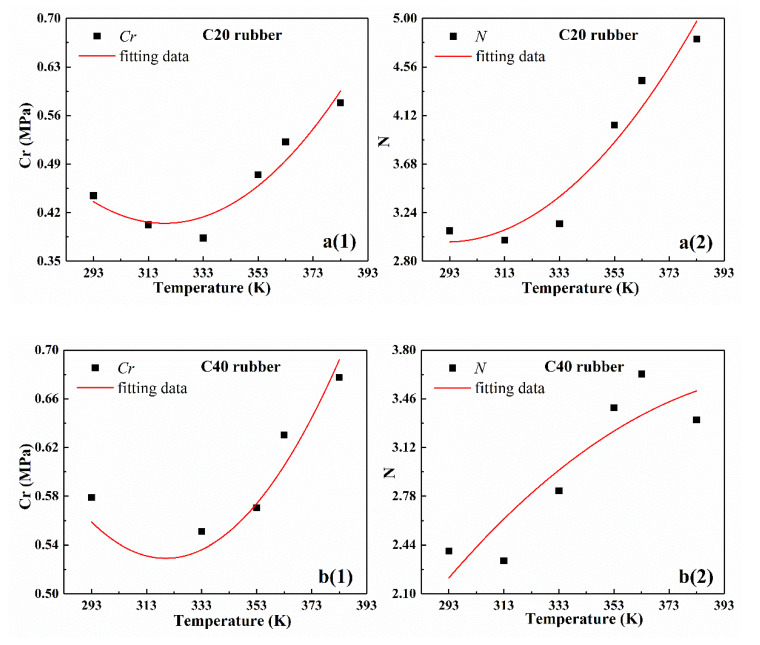

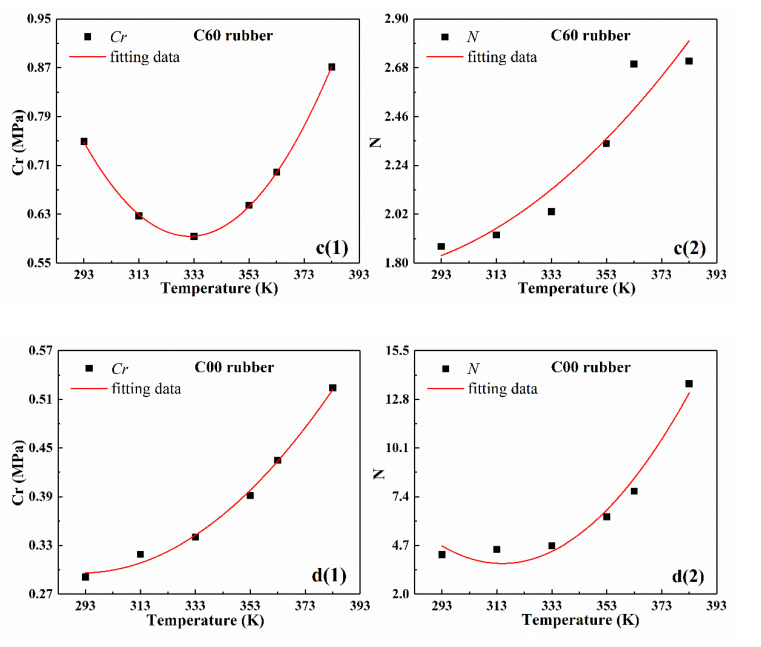
Temperature-dependence curves of the eight-chain model parameters of four different types of rubber and the corresponding fitting curves with quadratic function. (**a1**–**d1**) are the fitting curves and experimental data of parameter *Cr.* (**a2**–**d2**) are the fitting curves and experimental data of parameter *N.*

The test results of rubber specimens were used as an example to describe the analysis process of the temperature-dependence clearly. The relationship between the parameters and the temperature is given in Table 2 and is shown in Figure 5. From Figure 5, as the temperature increased, the two parameters of the eight-chain model showed different trends. By analyzing the trends based on the characteristics of the graph and the physical meaning of the parameters, the following conclusions can be obtained:

(1) Based on the variation of model parameter curves with temperature in Figure 5 and the simplicity of the model, the quadratic function relationship was suitable to describe the trend of *Cr* with temperature.

(2) According to Arruda-Boyce’s eight-chain theory, the second parameter *N* in the eight-chain model is a temperature-independent constant. However, according to literatures [36,37], *N* was actually temperature-dependent. From Figure 5, as the temperature increased, *N* changed approximately in the form of a quadratic function.

According to Figure 5 and the correlation coefficients in Table 3, the numerical fitting results using quadratic function were basically consistent with the experimental data. Thus, the numerical fitting with quadratic function can fully meet the accuracy requirements of practical application.

Therefore, the relationship between material parameters and temperature in the eight-chain model can be quantitatively expressed as follows.

(7) $$\{\begin{array}{c} Cr=A_{0}+A_{1}T+A_{2}T^{2}\\ N=B_{0}+B_{1}T+B_{2}T^{2} \end{array}$$

where *A*_0_, *A*_1_, *A*_2_, *B*_0_, *B*_1_, and *B*_2_ are the temperature-dependent characterization parameters of the eight-chain model, which can be obtained by fitting the parameters of the eight-chain model. For the same filled rubber with CB content, the temperature-dependent characterization parameters were obtained by fitting the eight-chain model parameters at different temperatures using Equation (7), as shown in Table 3.

Furthermore, combined with Equations (3) and (7), the eight-chain model with explicit temperature parameters can be obtained as follows:

(8) $$\{\begin{array}{c} W_{8}=nk\theta \sqrt{N}\left[\beta \lambda_{chain}+\sqrt{N}\ln\left(\frac{\beta }{\sinh\beta }\right)\right]\\ Cr=A_{0}+A_{1}T+A_{2}T^{2}\\ N=B_{0}+B_{1}T+B_{2}T^{2} \end{array}$$

**Figure 6:**
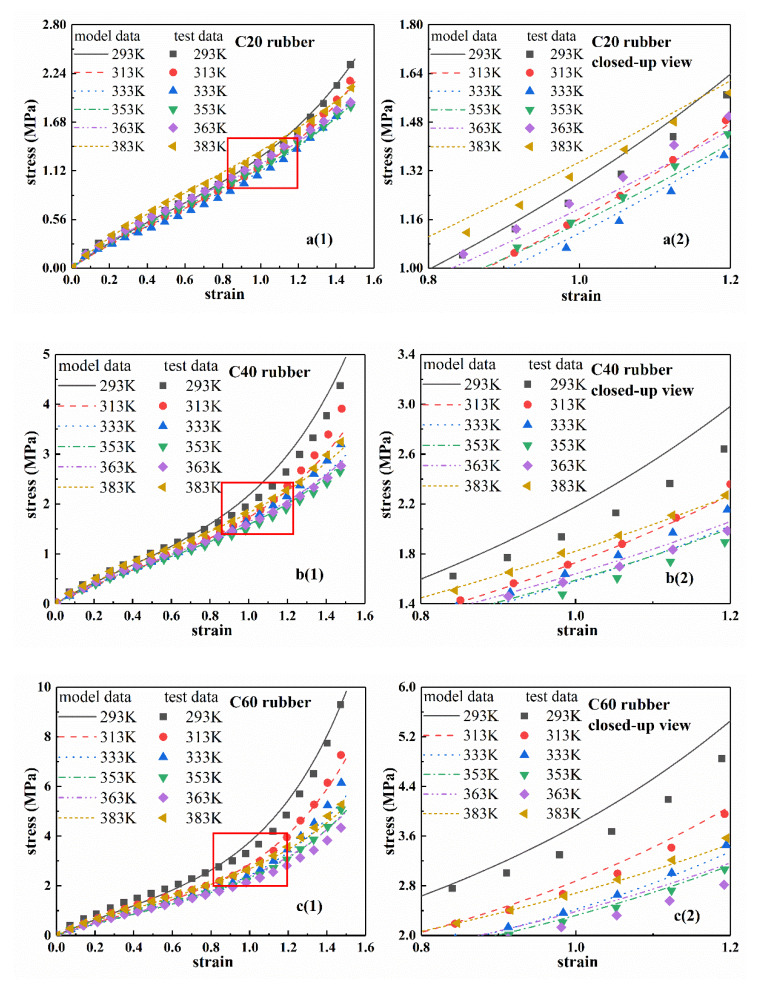

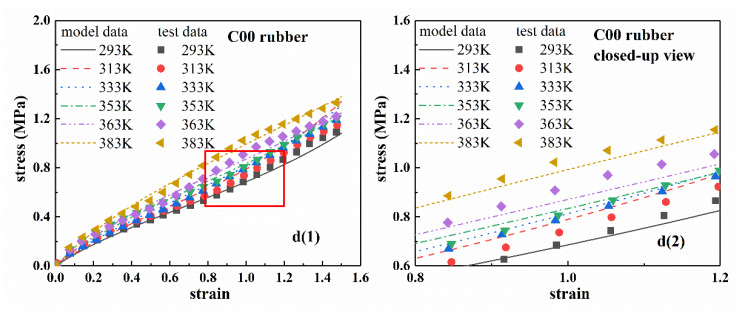
Model stress–strain curves of four types of rubber at different temperatures based on the Equations (8). (**a1**–**d1**) are the model curves and experimental data of four types of rubber; (**a2**–**d2**) are the corresponding local closed-up views.

From Equation (8) and the parameters of Table 3, the stress–strain curves of four types of rubber at a certain temperature can be obtained, which are called the ‘model curves’ of the eight-chain model with explicit temperature parameters. The ‘model curves’ are shown in Figure 6 together with the closed-up views. Figure 6a2–d2 are the closed-up views. From Figure 6, the stress–strain curves of the eight-chain model with explicit temperature parameters agreed well with the experimental results. Therefore, the eight-chain model with explicit temperature parameters can accurately describe the temperature-dependence of hyperelastic mechanical behavior for rubber materials.

According to Equation (8), in the temperature-dependent model, by setting the nominal strain to a constant value, the relationship between stress and temperature under constant strain can be obtained. Figure 2 shows the stress–temperature model curves for three types of filled rubber at strain *ε* = 0.3, 0.6, 0.9, and 1.2. From Figure 2, under constant strain, the stress–temperature curves of the improved eight-chain model with explicit temperature parameters agreed well with the experimental data. Therefore, the improved eight-chain model can accurately describe the trend of the stress of the filled rubber over temperature under a constant strain.

## 6. Application of Eight-Chain Model with Explicit Temperature Parameters in FEA

**Figure 7:**
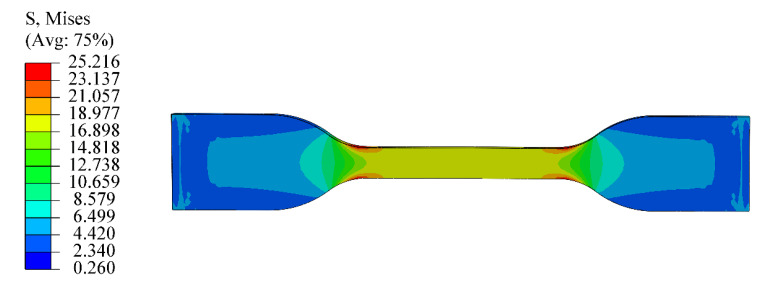
The stress contour picture of finite element analysis (FEA) of rubber specimen C60 at 273 K.

**Figure 8:**
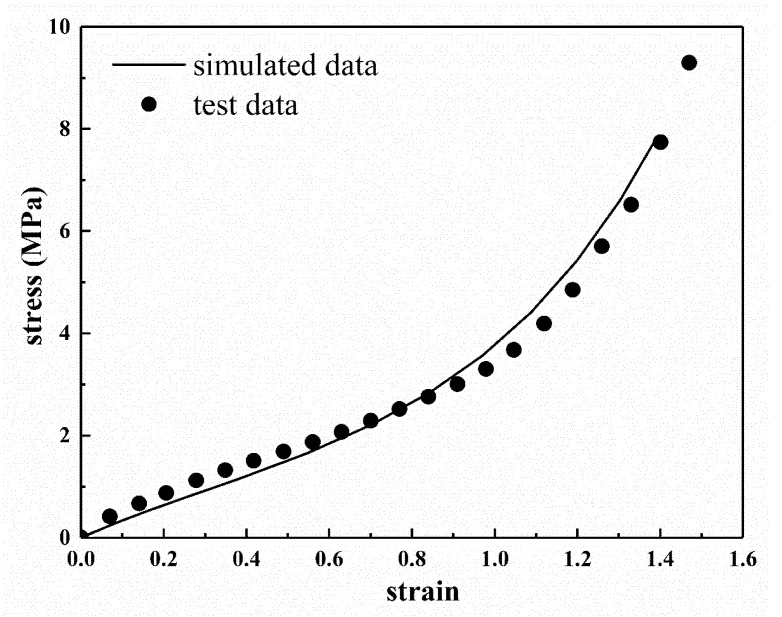
The stress–strain curves of the FEA results and experimental data of the rubber specimen C60.

According to the eight-chain model with explicit temperature parameters and Table 3, the model parameters were obtained, and then the parameters were applied to the finite element simulation. The following finite element simulation was carried out for the experimental data of sample C60 at 293 K. The corresponding values of *Cr* and *N* were obtained according to Table 3 and Equation (8), and then the FEA of uniaxial tension was carried out using ABAQUS. Figure 7 shows the stress contour picture under uniaxial tension analysis. We can clearly see that the stress distribution in the middle part is homogeneous. Moreover, the stress–strain curve of the tensile is obtained from the homogeneous deformation region in the middle, as shown in Figure 8. It can be seen from Figure 8 that the simulated tensile data are in good agreement with the experimental data. From the comparison results, it is obvious that the eight-chain model with explicit temperature parameters has a good ability to predict the uniaxial tensile test data and can be well applied to the actual working conditions as it can fully ensure the accuracy requirements. Furthermore, it can be concluded that the eight-chain model with explicit temperature parameters has good engineering practicability.

According to the eight-chain model with explicit temperature parameters, as long as the temperature of the rubber specimen is known, the corresponding model parameters can be calculated immediately, so the improved eight-chain model can be quickly applied to FEA. The improved eight-chain model can well avoid the parameter instability of the eight-chain model caused by experimental data fitting and provides a more convenient and accurate method for the FEA of various other finite element models. There is still a small deviation between the simulation results of the improved eight-chain model and the experimental data to a certain extent, indicating that the model still has room for improvement.

## 7. Discussion

An eight-chain model with explicit temperature parameters was developed based on the Arruda-Boyce model of the network constitutive Equation. The Arruda-Boyce model has a stable form of strain energy density function and has been proved to be feasible, not only under uniaxial tension, but also under other deformation states. Therefore, it is also possible to extend the application of the improved eight-chain model with explicit temperature parameters to multiaxial stress-strain state.

## 8. Conclusions

Based on the experimental data of rubber samples, the following conclusions can be obtained:

(1) The hyperelastic mechanical behavior of carbon black filled rubber was dependent on temperature in a relatively large deformation range. Based on the stress-strain curves, compared with unfilled rubber, the filled rubber exhibited more complex mechanical characteristics with the change of temperature. In addition, the filling amount of carbon black also had a great influence on the mechanical properties of rubber.

(2) The ability of the eight-chain model to characterize the dependence of rubber elastic mechanical behavior on temperature was analyzed. The fitting stress-strain curves of the rubber obtained by the eight-chain model at different temperatures were in a good agreement with the experimental results. Therefore, the eight-chain model can accurately characterize the temperature-dependent elastic mechanical behavior of rubber in a certain deformation range.

(3) Based on the eight-chain model and the phenomenological theory, an improved eight-chain model with explicit temperature parameters was developed. The model with explicit temperature parameters can well describe the elastic mechanical behavior of rubber at different temperatures, which can further expand the application scope and ability of the eight-chain model.

(4) The eight-chain model with explicit temperature parameters is used to FEA. The comparison between the simulation results and the test data verified the rationality of the model.

## Figures and Tables

**Table 1:** Formulas and codes of four kinds of carbon black filled vulcanized rubber (UNIT: PHR).

Code	C00	C20	C40	C60
NR^1^	100	100	100	100
CB^2^ N234	0	20	40	60
Zinc oxide	5	5	5	5
Stearic acid	3	3	3	3
Sulfur	2	2	2	2
Accelerator NS	1	1	1	1
Antioxidant 4020	1.2	1.2	1.2	1.2
Total	112.2	132.2	152.2	172.2

^1^NR, natural rubber; ^2^CB, carbon black.

**Table 2:** Model parameters of four types of rubber at different temperatures.

Temperature (K)	C00		C20		C40		C60	
	*Cr*	*N*	*Cr*	*N*	*Cr*	*N*	*Cr*	*N*
293	0.29118	4.19267	0.44451	3.07656	0.57884	2.39836	0.74927	1.87504
313	0.31922	4.48929	0.40255	2.99207	0.48903	2.33111	0.62706	1.92656
333	0.34026	4.67243	0.3835	3.14015	0.55098	2.81744	0.59364	2.03099
353	0.34248	4.38657	0.47456	4.03382	0.57048	3.39614	0.64449	2.33785
363	0.36903	4.54359	0.52195	4.43646	0.63029	3.63281	0.69873	2.69641
383	0.39834	4.29581	0.57828	4.81267	0.6774	3.31172	0.87105	2.71005

**Table 3:** Temperature-dependent characterization parameters of the four types of rubber.

Code	*A*_0_	*A*_1_	*A*_2_	Value R	*B*_0_	*B*_1_	*B*_2_	Value R
C00	0.3057	−0.00101	2.71 × 10^−5^	0.9973	7.27996	−0.17078	0.00204	0.9855
C20	0.50255	−0.00426	4.64 × 10^−5^	0.9568	3.05639	−0.00888	2.39 × 10^−4^	0.9691
C40	0.50255	−0.00384	4.10 × 10^−5^	0.92004	1.7326	0.02563	−8.57 × 10^−5^	0.8978
C60	0.95029	−0.0122	1.04 × 10^−4^	0.99989	1.76326	0.00219	6.59 × 10^−5^	0.9550
